# Supplementary material for: Fructose overconsumption impairs hepatic manganese homeostasis and ammonia disposal
Source: Nat Commun. 2023 Dec 1;14:7934. doi: 10.1038/s41467-023-43609-0 (PMC10692208; doi:10.1038/s41467-023-43609-0)

Fructose Overconsumption Impairs Hepatic Manganese Homeostasis and Ammonia Disposal

Supplementary data

Supplementary Tables: 4

Supplementary Figures: 9

Uncropped blot scans in Supplementary figure 6c

Supplementary Table 1. Primer sequence for mouse genotyping.

Genes	Primers	Sequence (5'-3')
LacZ	Forward	GAGCTGGGTAATAAGCGTTGGCAATT
	Reverse	GCAGTAAGGCGGTCGGGATAGTTT
Flp	Forward	CACTGATATTGTAAGTAGTTTGC
	Reverse	CTAGTGCGAAGTAGTGATCAGG
Slc30a10^{flox}	Forward	CCAAGGGAGCAGGGACAGACT
	Reverse	TGAGGGGAAGAGAAGGAAACATAA
Cre	Forward	AGATGGCGCGGCAACACC
	Reverse	GCGGATCCGAAAAGAAAA

Supplementary Table 2. Primer sequence for RT-PCR analysis.

Genes	Primers	Sequence (5'-3')
36b4	Forward	TGGTTGCTTTGGCGGGATTAGTCG
	Reverse	AAGCGCGTCCTGGCATTGTCTGTG
Chrebpβ	Forward	TCTGCAGATCGCGTGGAG
	Reverse	CTTGTCCCGGCATAGCAAC
Slc11a2	Forward	ACGGGGCCTGGCTTTCTTATG
	Reverse	CACCGCCTGCTCCACCTGA
Slc13a5	Forward	ACCAGCGAGTTGCCAGGAAGC
	Reverse	GCATGAAGCCGGGGTCTCG
Slc39a8	Forward	GCGCCAACCGGAGCCTGTC
	Reverse	CCCAATAGCGAGTCCCACGAAATA
Slc39a14	Forward	GGAGTGGGCCGGGATAATGTTTC
	Reverse	GCGCTTGGCTTCCCCTCCTCT
Trf	Forward	CGTGGCGGCGGAGTTTTATGG
	Reverse	GCGGGTATTGTCAAGGCAGAGCAG
Tfrc	Forward	CATACTCAGTTTCCGCCATCTCAG
	Reverse	GACTTCGCCGCAACACCAG
Slc30a10	Forward	AGGGCGCGACCGTGTTCTCT
	Reverse	TATTCCGTGCCCTTCTGGTGTTTG
Slc40a1	Forward	TGTCCCCAACTACCAAAATACCTG
	Reverse	CTGGGCAAATCGGAAATACATAAG
Atp2c1	Forward	CTTGGCCTGGTCGGAATCAT
	Reverse	GTCTGGCCCATCGCAACTC
Fgf21	Forward	CAAGCATACCCCATCCCTGACTCC
	Reverse	CTGCGCCTACCACTGTTCCATCCT
Khk	Forward	CCCACCGCCCCGAGTAGTAGACAC
	Reverse	CACACCTGCCGGGGAATGG
Pklr	Forward	GGACAAGGGGCGATGCAAAGACAG
	Reverse	ACCCCGAAGCGCAGATCCAAAAGA
Acc1	Forward	CTTCGGGGTGGTTCTTGGGTTGTG
	Reverse	CCTGCATCCGGCCTGGTGTG
Acc2	Forward	CGGAGGGCACGGTGGAGATTA
	Reverse	CATGTGGCCCGGGGTGTCGTG
Fasn	Forward	AAGTTGCCCGAGTCAGAGAA
	Reverse	CGTCGAACTTGGAGAGATCC
Vegf	Forward	CTCGCGGGATTGCACGGAAACTT
	Reverse	GCGCGGCTGGAGCACTGTCT

Supplementary Table 3. Information about the primary antibodies

Antibody	Company	Catalogue	Usage	Wording dilution
anti-β-actin	Proteintech	60008-1-Ig	WB	1:5000
anti-ChREBP	Santa Cruz	sc-33764	WB	1:500
anti-ARG1	Proteintech	16001-1-AP	WB	1:1000
anti-Cu/Zn-SOD	Proteintech	10269-1-AP	WB	1:1000
anti-Mn-SOD	Proteintech	24127-1-AP	WB	1:1000
anti-FLAG	Abmart	M2008	WB/IHC	1:5000 for WB<br>1:500 for IHC
anti-MDR1	Novus	SN06-42	IHC	1:200
anti-HIF1α	Invitrogen	PA3-16521	WB	1:1000
anti-HIF2α	Abcam	Ab109616	WB	1:1000

Supplementary Table 4. Primer sequence used to amplify *Slc30a10* gene in ChIP analysis.

Primer set	Primers	Sequence (5’-3’)	Position*
Set 1	Forward	GGCTCCCCCATCCACACC	-1521
	Reverse	TTCCAGCCCCAGCAAGCAGCAACT	-1237
Set 2	Forward	GCTGCCTGCCTTCCTCTCCTCTGT	-511
	Reverse	CTCCCGTTAACCCCTGTCCCTGAA	-347

*Position relative to transcription start site (+1) in bp

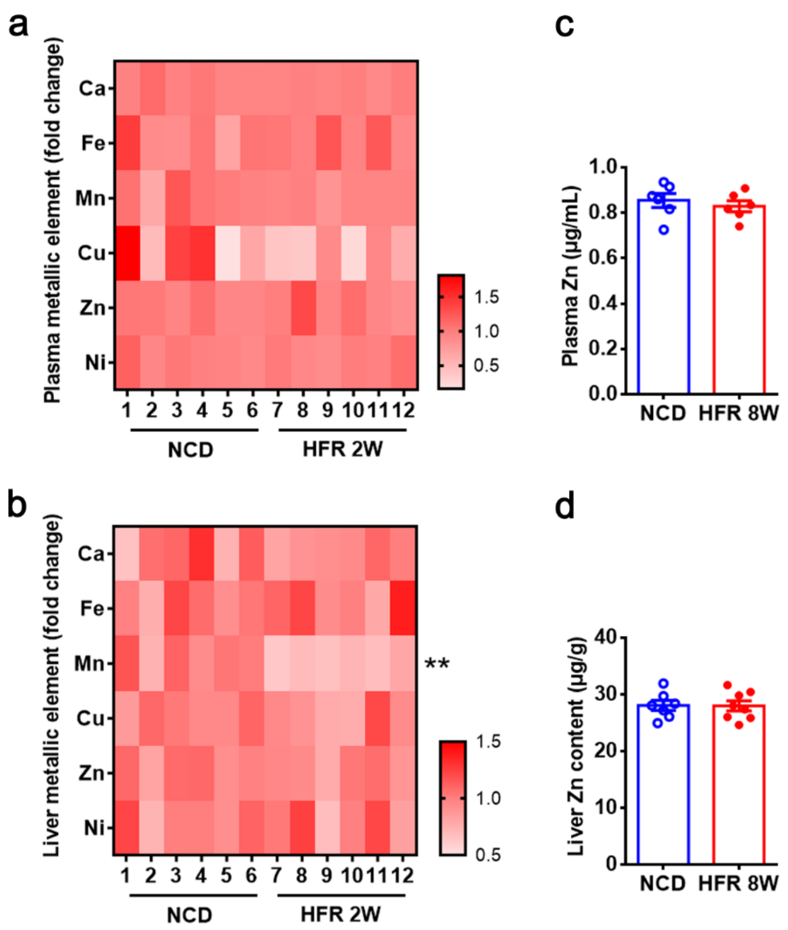

Supplementary Fig. 1. Analysis of metallic elements in plasma and liver. C57BL/6J male mice at the age of 2 months were fed normal chow diet (NCD) or a conventional high fructose diet (HFR, 65% fructose, Research Diets) for 2 weeks (**a-b**) or 8 weeks (**c-d**) (n=6~8). **a-b** Fold changes of Ca, Fe, Mn, Cu, Zn, Ni in the plasma (**a**) and liver (**b**). **c-d** Mn content in the plasma (**c**) and liver (**d**) from the mice fed HFR for 8 weeks. Data represent means±SEM. Two-tailed unpaired Student's *t*-test. **P<0.01.

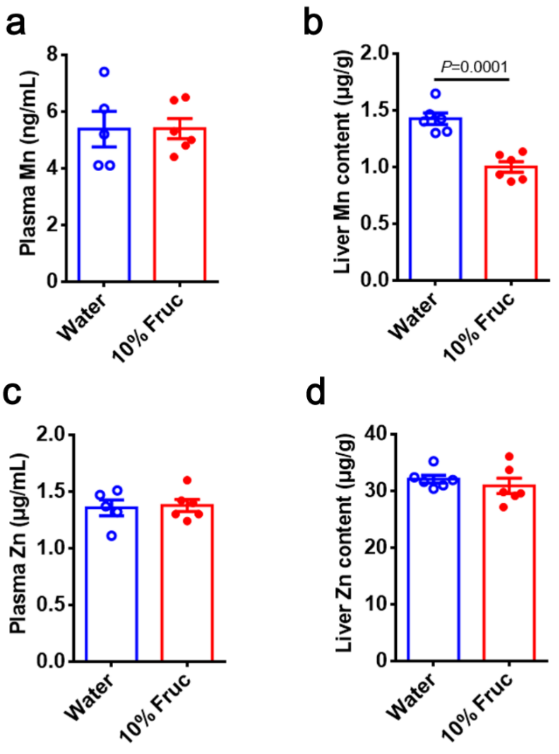

Supplementary Fig. 2. Fructose water intake reduces liver manganese content in mice.

C57BL/6J male mice at the age of 2 months were subjected to 10% fructose drink (10% Fruc) or tap water (Water) for 2 weeks (n=5~6). Their Mn content in the plasma (**a**) and liver (**b**), Zn content in the plasma (**c**) and liver (**d**). Data represent means±SEM. Two-tailed unpaired Student's *t*-test.

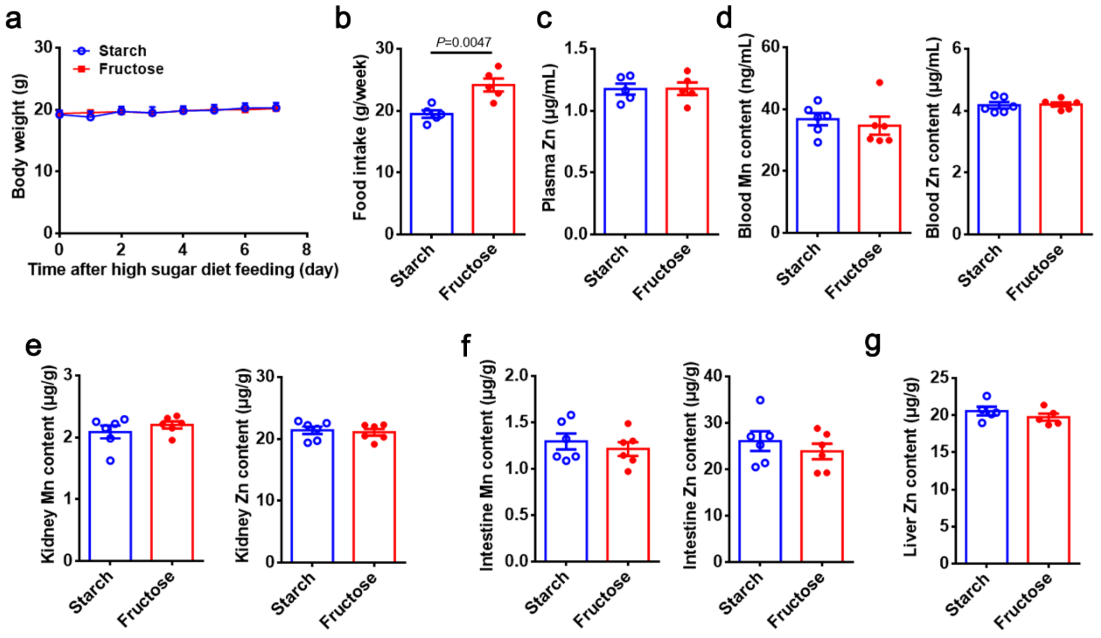

Supplementary Fig. 3. High fructose diet does not change Mn contents in blood and extrahepatic tissues.

C57BL/6J male mice at the age of 2 months were fed high starch diet (60% starch) or high fructose diet (60% fructose) for 1 week (n=5~7). **a** The body weight. **b** Food intake. **c** Plasma Zn content. **d** Blood Mn content and Blood Zn content. **e** Kidney Mn content and Kidney Zn content. **f** Intestine Mn content and Intestine Zn content. **g** Liver Mn content. Data represent means±SEM. Two-tailed unpaired Student's *t*-test.

a

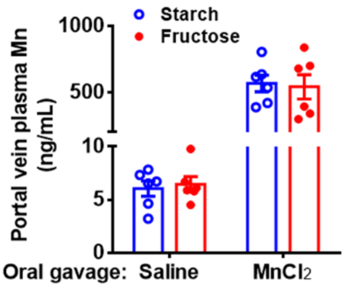

Supplementary Fig. 4. Fructose overconsumption does not affect intestinal manganese absorption.

C57BL/6J male mice at the age of 2 months were fed high starch diet (60% starch) or high fructose diet (60% fructose) for 1 week (n=6). Portal vein plasma was collected 30 minutes after oral gavage with saline or manganese chloride (72 mg/kg $MnCl_2 \cdot 4H_2O$). Data represent means±SEM. Two-tailed unpaired Student's *t*-test.

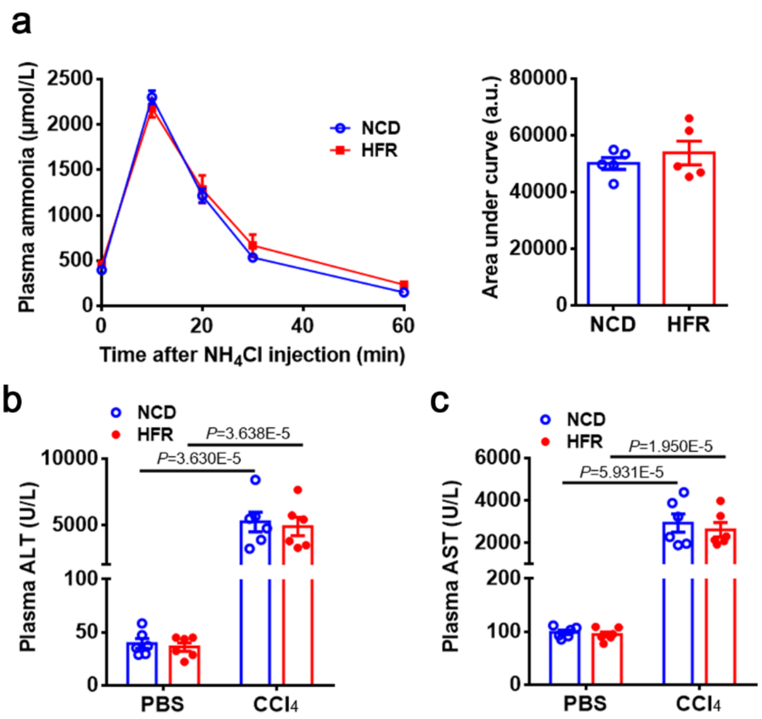

Supplementary Fig. 5. Fructose overconsumption does not affect blood ammonia clearance in mice with normal liver function.

C57BL/6J male mice at the age of 2 months were fed normal chow diet (NCD) or high fructose diet (HFR, 65% fructose) for 2 weeks (n=5~6 mice each group). **a** Plasma ammonia levels at the indicated time points after NH_4Cl injection (4 mmol/kg body weight) with area under curve. **b-c** NCD-fed or HFR-fed mice were i.p. injected with CCl_4 (1 ml/kg body weight) or PBS. Two days later, liver injury was indicated by elevated plasma ALT (**b**) and AST levels (**c**). Data represent means±SEM. Two-tailed unpaired Student's *t*-test.

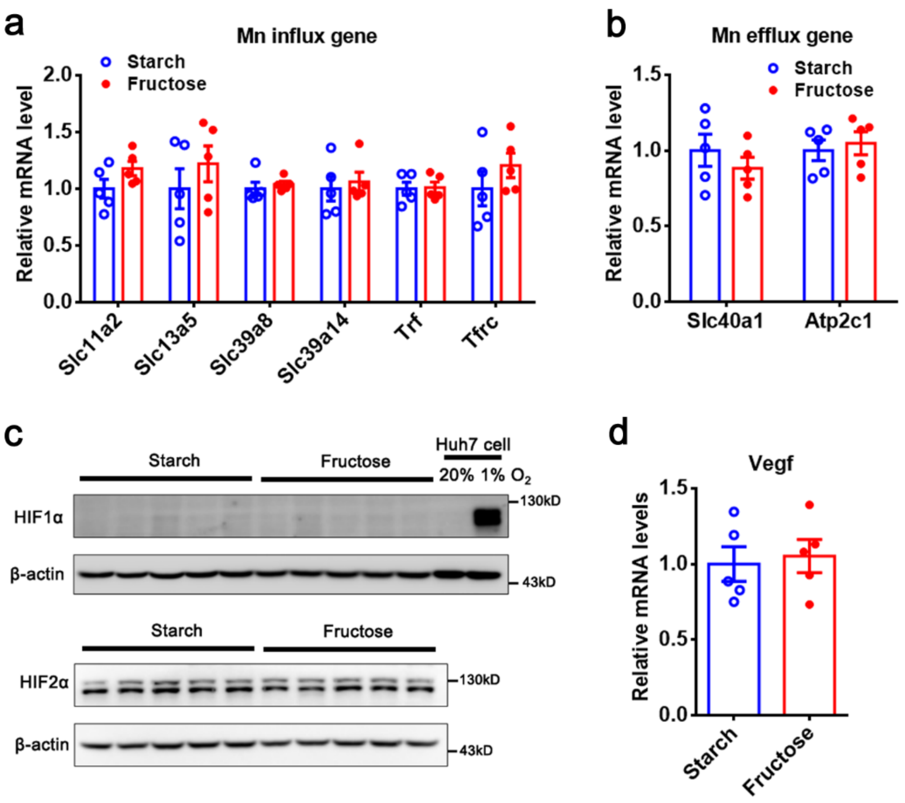

Supplementary Fig. 6. Fructose overconsumption does not affect the expression of HIF and other Mn transporter genes than Slc30a10.

C57BL/6J male mice were fed 60% starch or 60% fructose diets for 1week (n=5). **a-b** Liver mRNA levels of Mn exporter genes (**a**) and importers genes (**b**). **c** Protein expression of HIF1α and HIF2α in the liver whole lysate analyzed by Western blot and β-actin as a loading control. Huh7 cells were cultured at 20% O_2 or 1% O_2 for 12h before harvested. **d** Liver *Vegf* mRNA levels. Data represent means±SEM. Two-tailed unpaired Student's *t*-test.

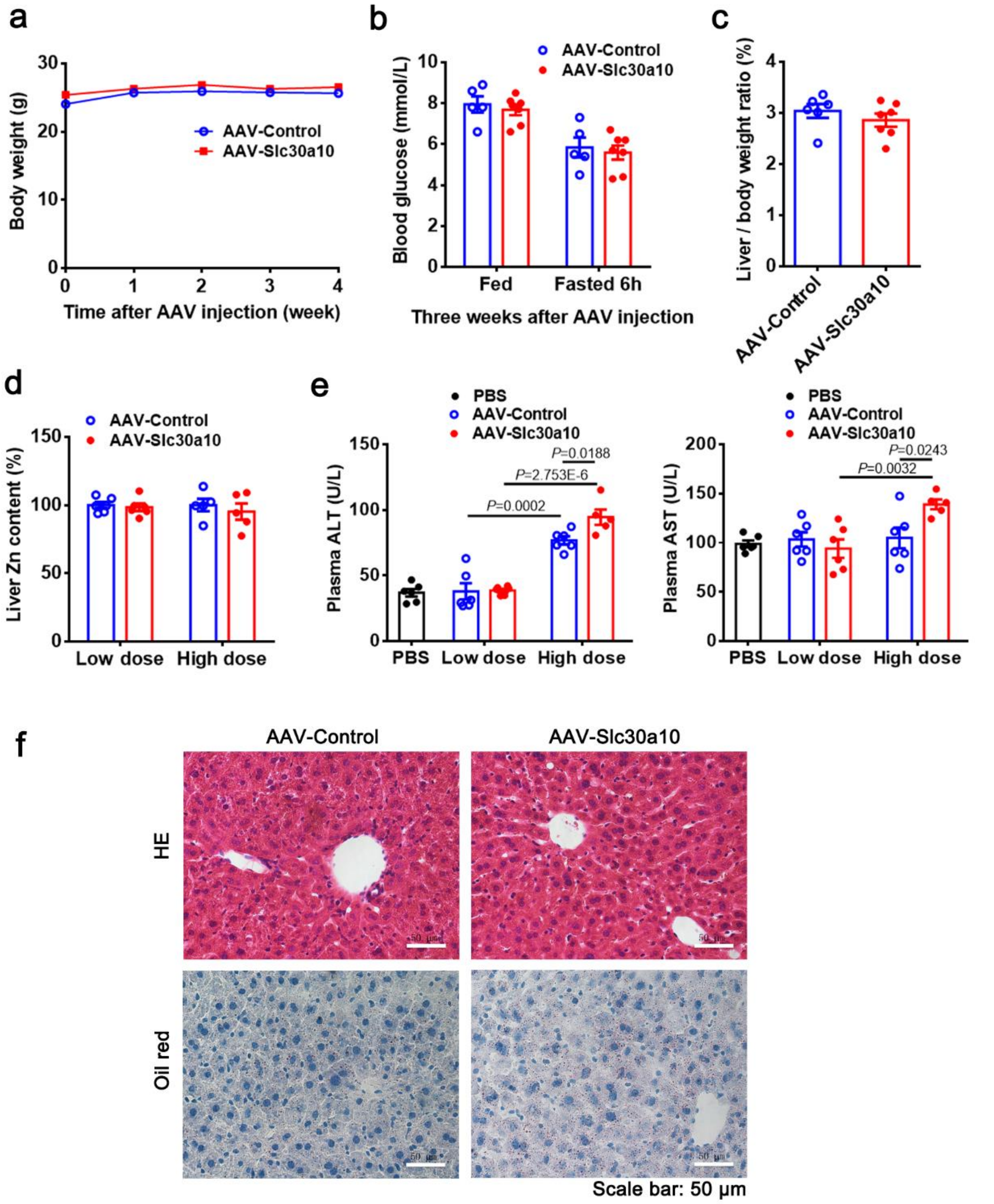

Supplementary Fig. 7. Hepatic overexpression of Slc30a10 per se does not affect blood glucose levels and hepatic lipid accumulation.

Two-month-old C57BL/6J mice were intravenously injected with AAV expressing Flag-Slc30a10 (AAV-Slc30a10) or AAV-Control at low dose (2×10^{10} vg/ mice) and high dose (2×10^{11} vg/ mice), and fed NCD for 4 weeks before sacrificed (n=5~7). **a** The body weight. **b** Blood glucose in the fed state or fasted 6h state. **c** Liver weight to body weight ratio. **d** Liver Zn content. **e** Plasma ALT and AST levels. **f** H-E staining and oil red staining of liver sections. Scale bar: 50 μm. Data represent means±SEM. Two-tailed unpaired Student's *t*-test.

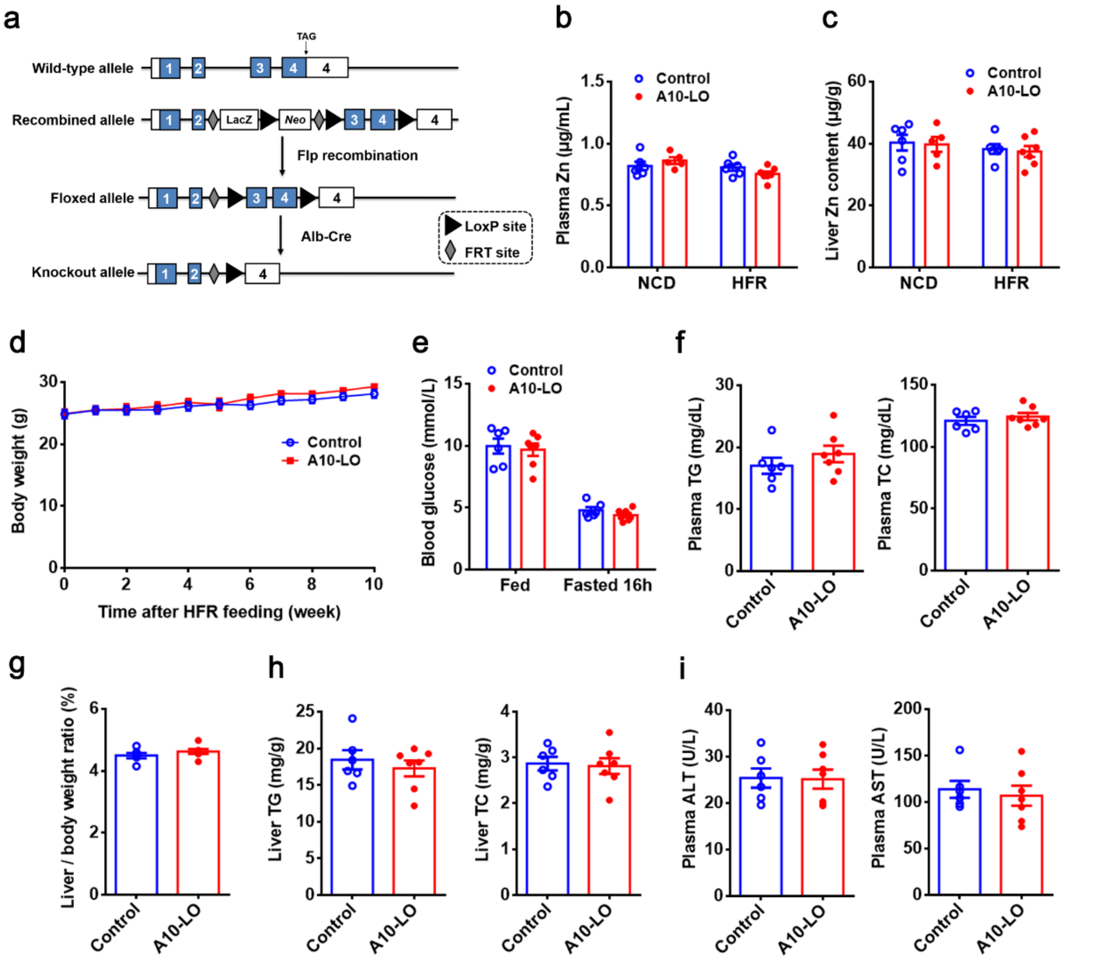

Supplementary Fig. 8: Generation and characterization of Slc30a10 liver-specific knockout mice.

a Schematic demonstration for the generation of Slc30a10 liver-specific knockout (A10-LO) mice. **b-c** Control and A10-LO mice were fed normal chow diet (NCD) or high fructose diet (HFR) for 2 weeks (n=5~7). Plasma Zn levels (**b**) and liver Zn content (**c**). **d-i** Control and A10-LO mice were fed HFR for 10 weeks (n=6~7). Body weight (**d**), blood glucose in the fed state or fasted 16h state (**e**), plasma TG and TC levels (**f**), liver weight to body weight ratio (**g**), liver TG and TC content (**h**), and plasma ALT and AST levels (**i**). Data represent means±SEM. Two-tailed unpaired Student's *t*-test.

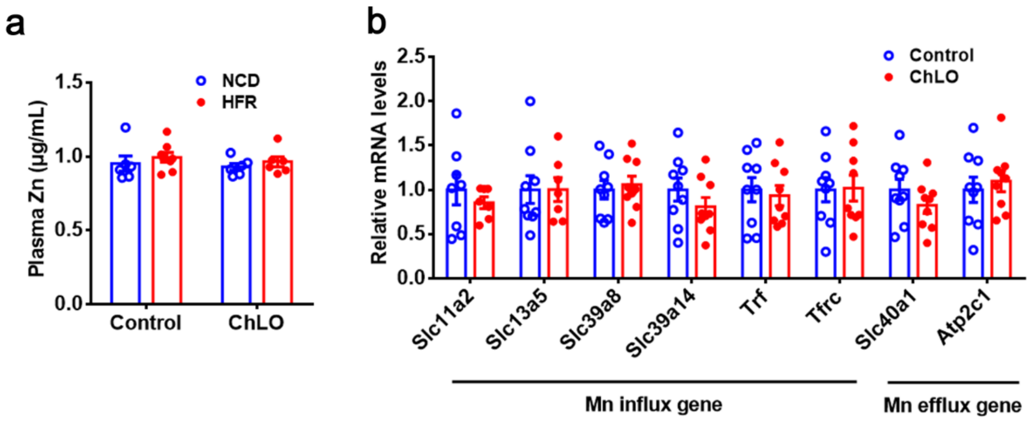

Supplementary Fig. 9. Hepatic ChREBP knockout does not affect the expression of Mn transporter genes other than Slc30a10.

Control and ChREBP liver-specific knockout (ChLO) male mice were fed NCD or HFR for 2 weeks (n=5~8). **a** Plasma Zn levels. **b** Liver mRNA levels of Mn exporter genes and importers genes of mice fed NCD. Data represent means± SEM. Two-tailed unpaired Student's *t*-test.

Uncropped blot scans in Supplementary figure 6c

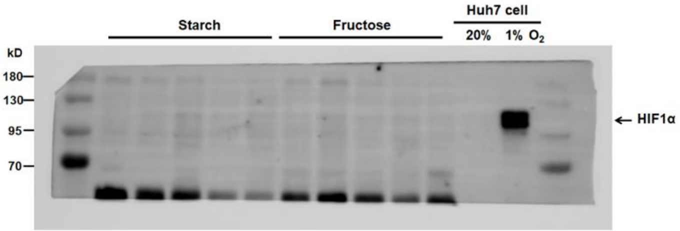

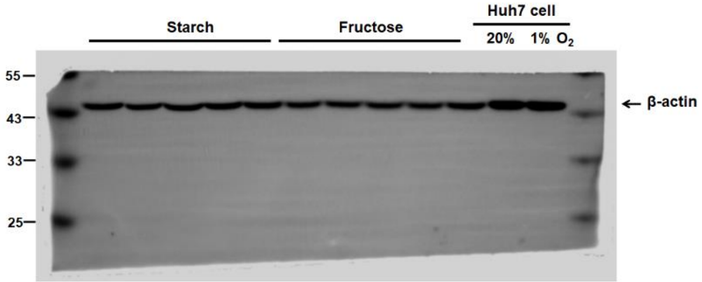

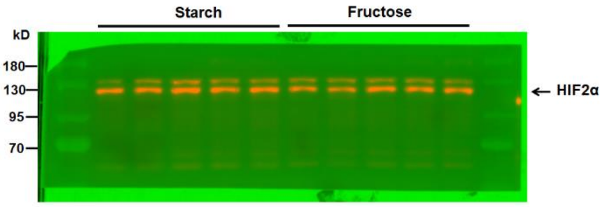

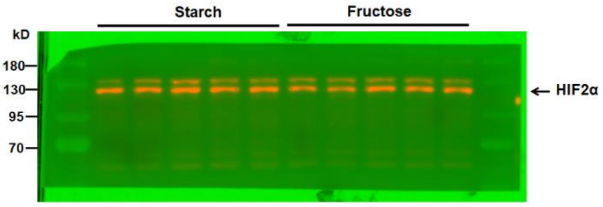

Supplement: Supplementary file 1 — Supplementary infomation [file 41467_2023_43609_MOESM1_ESM.pdf]
