# Supplementary material for: Fructose overconsumption impairs hepatic manganese homeostasis and ammonia disposal
Source: Nat Commun. 2023 Dec 1;14:7934. doi: 10.1038/s41467-023-43609-0 (PMC10692208; doi:10.1038/s41467-023-43609-0)
Supplement: Supplementary file 3 — Reporting Summary [file 41467_2023_43609_MOESM3_ESM.pdf]

nature portfolio

Corresponding author(s): Weiping Zhang
Last updated by author(s): 2023-11-2

Reporting Summary

Nature Portfolio wishes to improve the reproducibility of the work that we publish. This form provides structure for consistency and transparency in reporting. For further information on Nature Portfolio policies, see our Editorial Policies and the Editorial Policy Checklist.

Statistics

For all statistical analyses, confirm that the following items are present in the figure legend, table legend, main text, or Methods section.

n/a	Confirmed	
☐	☒	The exact sample size (n) for each experimental group/condition, given as a discrete number and unit of measurement
☐	☒	A statement on whether measurements were taken from distinct samples or whether the same sample was measured repeatedly
☐	☒	The statistical test(s) used AND whether they are one- or two-sided<br>*Only common tests should be described solely by name; describe more complex techniques in the Methods section.*
☐	☒	A description of all covariates tested
☐	☒	A description of any assumptions or corrections, such as tests of normality and adjustment for multiple comparisons
☐	☒	A full description of the statistical parameters including central tendency (e.g. means) or other basic estimates (e.g. regression coefficient) AND variation (e.g. standard deviation) or associated estimates of uncertainty (e.g. confidence intervals)
☐	☒	For null hypothesis testing, the test statistic (e.g. F, t, r) with confidence intervals, effect sizes, degrees of freedom and P value noted<br>*Give P values as exact values whenever suitable.*
☒	☐	For Bayesian analysis, information on the choice of priors and Markov chain Monte Carlo settings
☒	☐	For hierarchical and complex designs, identification of the appropriate level for tests and full reporting of outcomes
☒	☐	Estimates of effect sizes (e.g. Cohen's d, Pearson's r), indicating how they were calculated

Our web collection on statistics for biologists contains articles on many of the points above.

Software and code

Policy information about availability of computer code

Data collection	Images were collected using Olympus IX71 microscope with Andor Neo sCMOS camera, Olympus BX51 microscope.
Data analysis	Data analysis was performed with Graphpad Prism 8.4.0. Images were processed with Adobe photoshop CS6. Illustration was created with Biorender.com

For manuscripts utilizing custom algorithms or software that are central to the research but not yet described in published literature, software must be made available to editors and reviewers. We strongly encourage code deposition in a community repository (e.g. GitHub). See the Nature Portfolio guidelines for submitting code & software for further information.

Data

Policy information about availability of data

All manuscripts must include a data availability statement. This statement should provide the following information, where applicable:

- Accession codes, unique identifiers, or web links for publicly available datasets
- A description of any restrictions on data availability
- For clinical datasets or third party data, please ensure that the statement adheres to our policy

All data generated or analyzed during this study are included in this article (and its supplementary information files) , and archived in Figshare. They are available with no restrictions.

Research involving human participants, their data, or biological material

Policy information about studies with human participants or human data. See also policy information about sex, gender (identity/presentation), and sexual orientation and race, ethnicity and racism.

Reporting on sex and gender	All the experiments were performed on male mice. The conclusion is based on male mice which is indicated in the abstract.
Reporting on race, ethnicity, or other socially relevant groupings	no human subjects were included.
Population characteristics	N/A
Recruitment	N/A
Ethics oversight	Naval Medical University Animal Ethics Committee

Note that full information on the approval of the study protocol must also be provided in the manuscript.

Field-specific reporting

Please select the one below that is the best fit for your research. If you are not sure, read the appropriate sections before making your selection.

[x] Life sciences [ ] Behavioural & social sciences [ ] Ecological, evolutionary & environmental sciences

For a reference copy of the document with all sections, see nature.com/documents/nr-reporting-summary-flat.pdf

Life sciences study design

All studies must disclose on these points even when the disclosure is negative.

Sample size	Sample sizes were not predetermined with any statistical method and they were chosen depending on availability and general guideline to meet requirements for statistical analyses.
Data exclusions	No data were excluded for the analyses.
Replication	Reproducibility was confirmed. The number of independent experiments is described in the figure legends.
Randomization	In our experimental design, we ensured that similar numbers of mice or cells were randomly assigned to each group and subjected to each treatment that we tested.
Blinding	Investigators were blinded to group allocation during data collection. Mass spectrometry was performed by double-blinding.

Reporting for specific materials, systems and methods

We require information from authors about some types of materials, experimental systems and methods used in many studies. Here, indicate whether each material, system or method listed is relevant to your study. If you are not sure if a list item applies to your research, read the appropriate section before selecting a response.

Materials & experimental systems

n/a	Involved in the study	
[ ]	[x]	Antibodies
[ ]	[x]	Eukaryotic cell lines
[x]	[ ]	Palaeontology and archaeology
[ ]	[x]	Animals and other organisms
[x]	[ ]	Clinical data
[x]	[ ]	Dual use research of concern
[x]	[ ]	Plants

Methods

n/a	Involved in the study	
[x]	[ ]	ChIP-seq
[x]	[ ]	Flow cytometry
[x]	[ ]	MRI-based neuroimaging

Antibodies

Antibodies used	All antibodies used in this work were listed in supplementary table 3 with source information and catalog number.<br>The antibody anti-β-actin was purchased from Proteintech (60008-1-lg) used for WB with dilution 1:5000;<br>The antibody anti-ChREBP was purchased from Santa Cruz (sc-33764) used for WB with dilution 1:500;<br>The antibody anti-ARG1 was purchased from Proteintech (16001-1-AP) used for WB with dilution 1:1000;

	The antibody anti-anti-Cu/Zn-SOD was purchased from Proteintech (10269-1-AP) used for WB with dilution 1:1000;<br>The antibody anti-Mn-SOD was purchased from Proteintech (24127-1-AP) used for WB with dilution 1:1000;<br>The antibody anti-FLAG was purchased from Abmart (M2008) used for WB with dilution 1:5000 and usd for IHC with dilution 1:500;<br>The antibody anti-MDR1 was purchased from Novus (SN06-42) used for IHC with dilution 1:200;<br>The antibody anti-HIF1α was purchased from Invitrogen (PA3-16521) used for WB with dilution 1:1000;<br>The antibody anti-HIF2α was purchased from Abcam (Ab109616) used for WB with dilution 1:1000.
Validation	All commercially available primary antibodies have been validated by the manufactures (the information of Supplier and catalog number are included in Supplemental Table 3).

Eukaryotic cell lines

Policy information about cell lines and Sex and Gender in Research

Cell line source(s)	HEK293T (ATCC)
Authentication	The cell lines have been authenticated by STR profiling by the vendor. No further authentication was performed.
Mycoplasma contamination	Mycoplasma contamination was tested and found negative.
Commonly misidentified lines<br>(See ICLAC register)	No commonly misidentified cell lines were used.

Animals and other research organisms

Policy information about studies involving animals; ARRIVE guidelines recommended for reporting animal research, and Sex and Gender in Research

Laboratory animals	This study involved the use of the following strains of mice:<br>Slc30a10 liver-specific knockout mice (maintained on C57B6/J background). Adult male mice were used for experiments.<br>ChREBP liver-specific knockout mice (maintained on C57B6/J background). Adult male mice were used for experiments.<br>Alb-Cre transgenic mice (maintained on C57B6/J background). Adult male mice were used for experiments.
Wild animals	No wild animals were used.
Reporting on sex	All the experiments were performed on male mice, which is indicated in the Method.
Field-collected samples	No field-collected samples were used.
Ethics oversight	The study is ethically overseen by the Naval Medical University Animal Ethics Committee (Shanghai, China).

Note that full information on the approval of the study protocol must also be provided in the manuscript.
